# Supplementary figures and images for: Exploring the preservation of a parasitic trace in decapod crustaceans using finite elements analysis
Source: PLoS One. 2024 Apr 16;19(4):e0296146. doi: 10.1371/journal.pone.0296146 (PMC11020947; doi:10.1371/journal.pone.0296146)

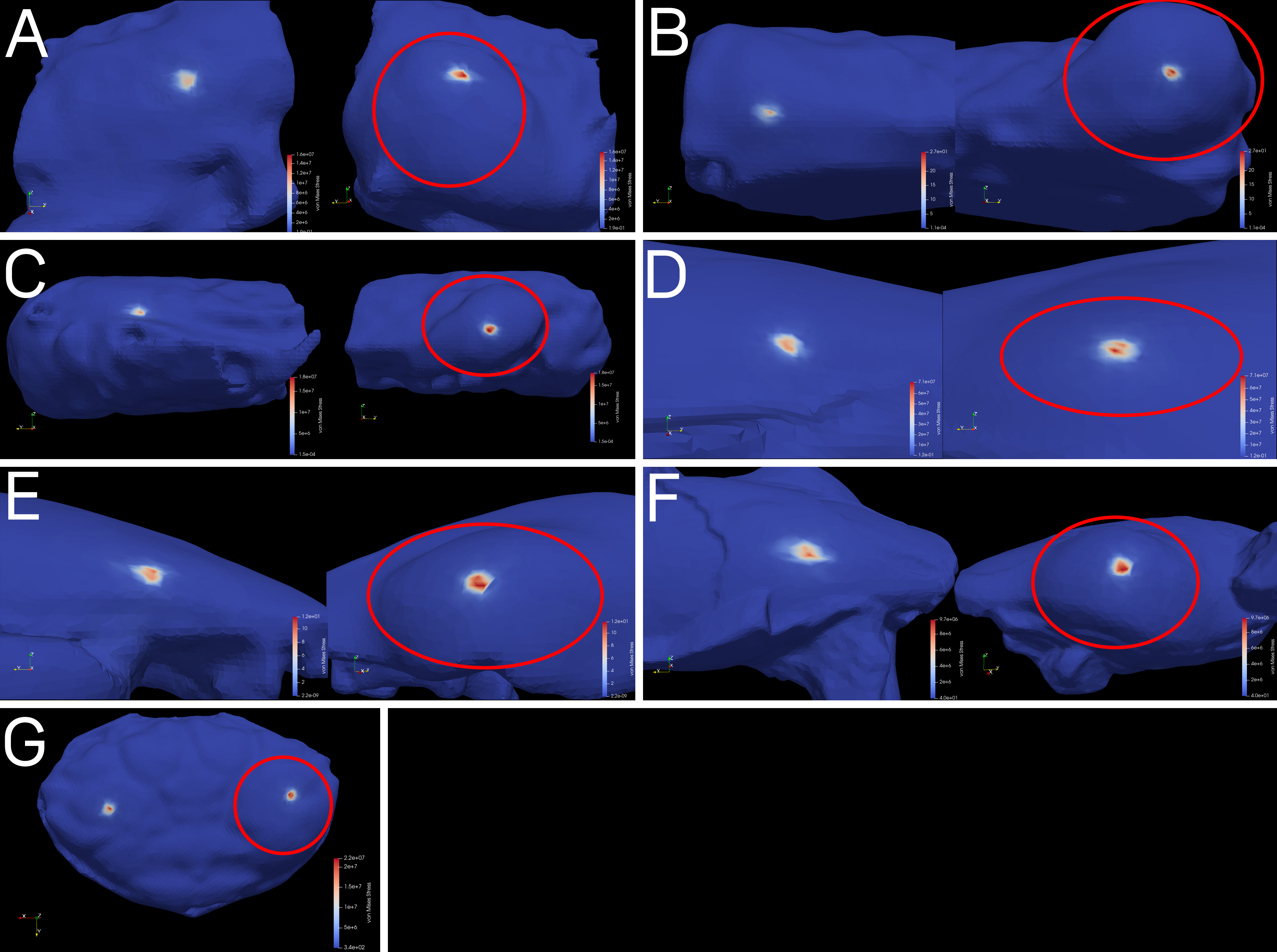

Supplement: S1 Fig — Swellings are displayed to the right and circled in red for each specimen. A-C) Modern specimens of Munida valida TAMU cat. no. 2–3061 (A,C), 2–3063 (B). D-F) Fossil specimens of Macroacaena rosenkrantzi NHMD MGUH 34322 (D), MGUH 34323 (E), MGUH 34324 (F). G) Fossil specimen of Panopeus nanus UF 288470. (TIF) [file pone.0296146.s001.tif]

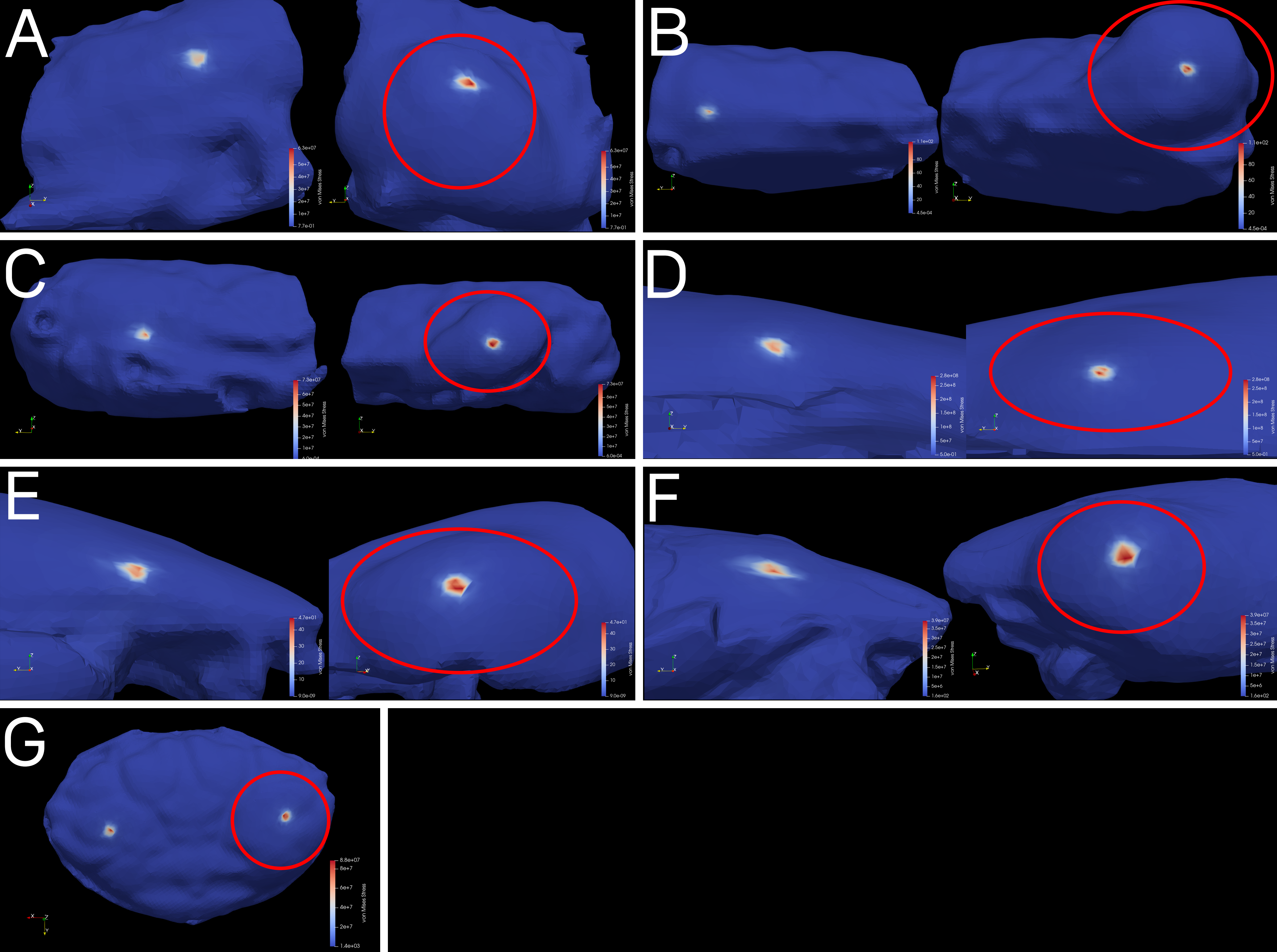

Supplement: S2 Fig — Swellings are displayed to the right and circled in red for each specimen. A-C) Modern specimens of Munida valida TAMU cat. no. 2–3061 (A,C), 2–3063 (B). D-F) Fossil specimens of Macroacaena rosenkrantzi NHMD MGUH 34322 (D), MGUH 34323 (E), MGUH 34324 (F). G) Fossil specimen of Panopeus nanus UF 288470. (TIF) [file pone.0296146.s002.tif]
